# Intrinsic electrical diversity of PV^+^ retinal ganglion cells

**DOI:** 10.1016/j.isci.2026.116842

**Published:** 2026-08-13

**Authors:** Viktória Király, Günther Zeck, Paul Werginz

**Affiliations:** 1 Institute of Biomedical Electronics, TU Wien, 1040 Vienna, Vienna, Austria

**Keywords:** retinal ganglion cells, spike generator, axon initial segment, action potential

## Abstract

Mouse retinal ganglion cells (RGCs) transform visual signals into spiking outputs and are known as a heterogeneous cell population. We combined electrophysiological recordings and anatomical analyses to characterize parvalbumin-positive (PV^+^) RGC subtypes, including ON-OFF direction selective (OODS), ON transient small receptive field (ON tr SmRF), OFF transient medium receptive field (OFF tr MeRF), and the OFF transient small receptive field (OFF tr SmRF) RGC. Intrinsic properties such as input resistance, maximum depolarization rate, and maximum rebound firing rate distinguished five functional subtypes, including one previously unmatched population. Analysis of axonal morphology revealed subtype-specific differences in axon initial segment (AIS) length and diameter. OFF tr MeRF RGCs display shortest AISs and smallest proximal axon diameter, correlating with unique near-threshold spikelets. Following axotomy, loss of the AIS reduced maximum firing rates and altered spike properties. Our results demonstrate substantial biophysical diversity among a subset of PV^+^ RGCs, complementing previously described differences.

## Highlights

• Distinct intrinsic electrical properties characterize PV^+^ retinal ganglion cells
• Cells with smallest axon diameters are prone to subtype-specific spikelet generation
• Loss of the axon initial segment reduces maximum firing rates and alters AP dynamics

## Graphical abstract

**Figure:**
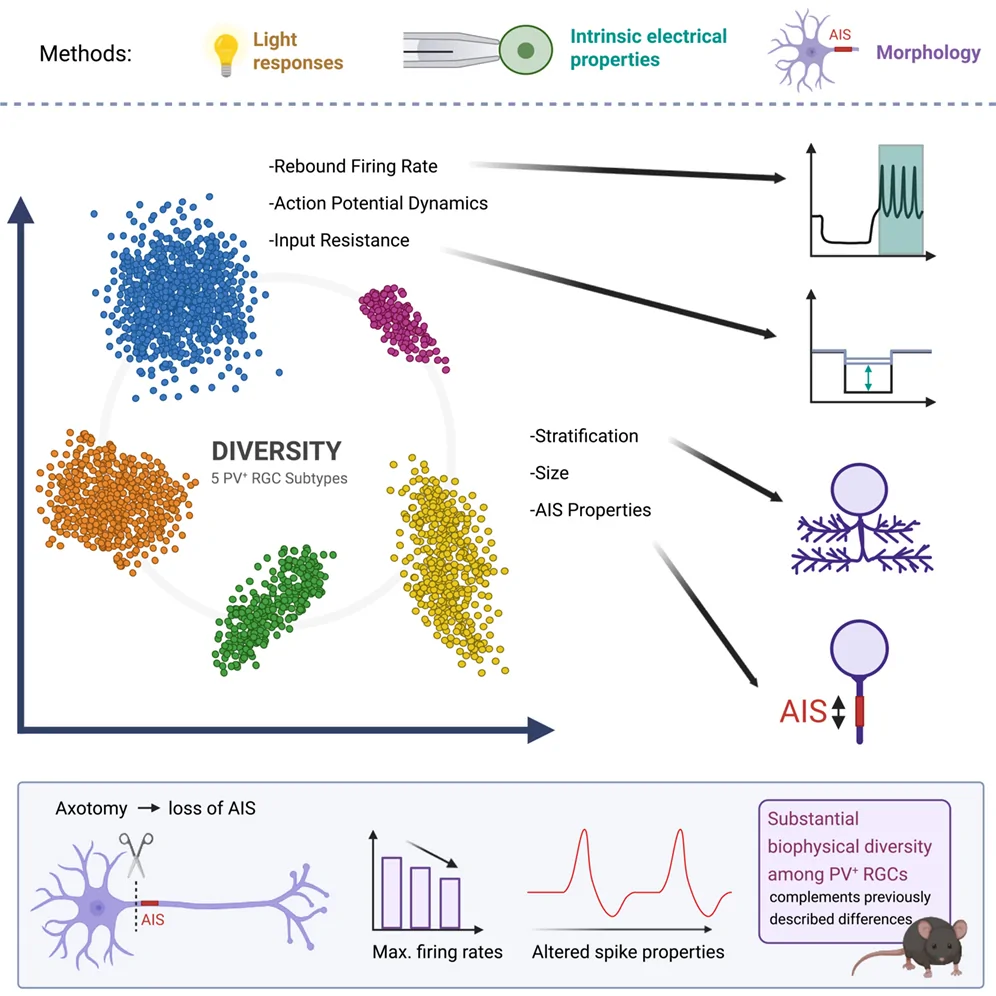

## Teaser

Biological sciences

## Introduction

Retinal ganglion cells (RGCs) are the sole output neurons of the retina, making the investigation of their anatomy and function crucial for understanding vision and vision-related diseases. RGCs form a highly diverse group of neurons within the retina. As technical advancements enabled recordings from larger neuron populations and more refined molecular tools became available, the field of retina research transitioned toward recognizing a much finer classification of RGC types. Numerous studies have attempted to classify mouse RGCs,^1^^,^^2^^,^^3^ yet a comprehensive dataset was only established following the work of Goetz et al.,^4^ which described a total of 42 RGC types based not only on anatomical and electrophysiological, but also transcriptional datasets.

Most studies have focused solely on retinal network analysis, i.e., the spiking output of RGCs in response to visual stimulation.^5^^,^^6^^,^^7^^,^^8^ However, RGC spiking output can be separated into a presynaptic component driven by bipolar and amacrine cell inputs as well as an intrinsic component, i.e., the spike generator, which both shape the final spiking output of RGCs. Among the first studies that have analyzed intrinsic electrical properties of RGCs, two representative examples focused on the cat^9^ and the rat retina.^10^ More recently, additional studies combined light stimulation and somatic current injections with anatomical reconstructions,^11^^,^^12^^,^^13^^,^^14^^,^^15^ which are essential to link functional and structural properties at the single-cell level. For example, our group provided a comprehensive analysis of the differential spiking output of mouse α RGCs.^13^ We were able to demonstrate that the intrinsic electrical properties in these cell types match their response profiles during visual stimulation, i.e., cells with more sustained responses to light stimulation also showed more sustained output during somatic current injections. Here, we aim to apply this approach to a subset of medium-sized soma parvalbumin-positive (PV^+^) RGCs (non-α), to achieve a more comprehensive understanding of their intrinsic electrical characteristics and their role in retinal signal processing. Such analyses are important, as only ∼5% of the total retinal cell population represents α RGCs.^16^

One cellular feature that has been identified as being crucial for spiking output is the axon initial segment (AIS) (reviewed in^17^). In RGCs, the AIS is located at the proximal axon ∼10–20 μm distant from the soma and has been attributed to be a key element for action potential generation and (back)propagation due to its expression of high levels of Nav1.6 sodium channels.^18^^,^^19^ For example, our group showed that the length of the AIS in OFF α transient RGCs is oriented along the dorsoventral axis, suggesting that longer AISs in dorsal cells are capable of sustaining prolonged spike trains.^14^ At the same time, ventral AISs are shorter and support transient spiking. Similar to these findings, Wienbar et al.^15^ demonstrated in Bursty Suppressed-by-Contrast (bSbC) RGCs that reduced AIS length, the composition of sodium channels, and lower sodium conductance can affect depolarization block, showing that even though these cells receive the same inputs as OFF α sustained RGCs, their intrinsic electrical properties are different, and that these differences affect output signals. Therefore, variations in AIS length, soma-AIS distance, and ion channel composition systematically vary with retinal position and cell size, and these features play a decisive role in shaping firing properties and output signals of RGCs. This highlights the AIS as a key adaptive structure linking cellular morphology to functional diversity in the retina.

In this study, we aimed to characterize the spiking output of a characterized subset of PV^+^ RGCs, focusing on their anatomy and intrinsic electrical properties, with particular attention to the proximal axon/AIS as a key determinant of action potential initiation and propagation.

## Results

We chose a PV^+^ mouse model to narrow down the more than 40 morphologically and functionally distinct RGC subtypes to approximately 8 types that express parvalbumin^20^; our goal was to investigate the intrinsic electrical properties of medium-sized soma RGCs.

### Experimenter-based identification of various PV^+^ RGC cell types

**Figure 1:**
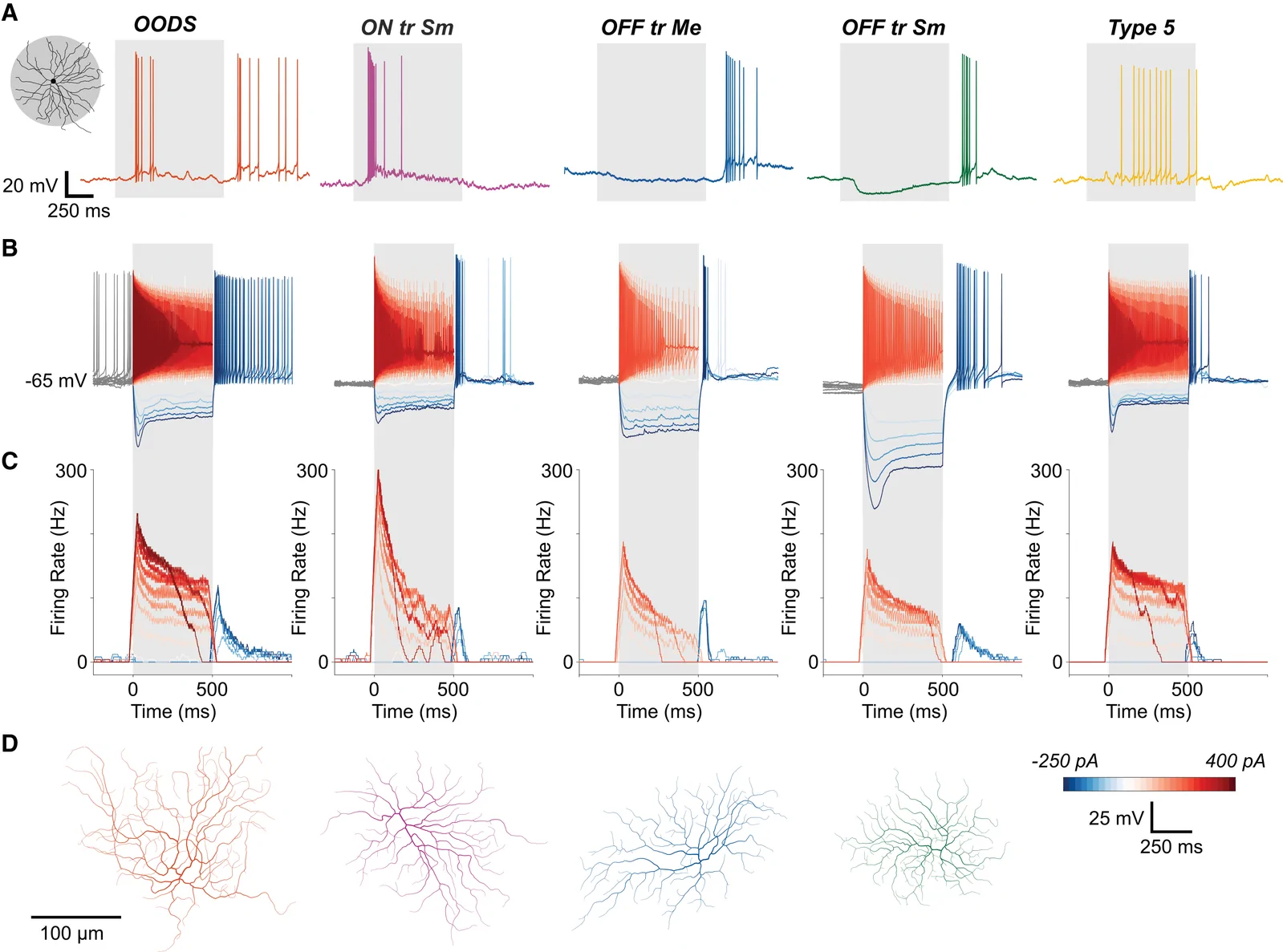
Intrinsic electrical and anatomical diversity in five types of PV^+^ RGCs (A) Representative light responses in five types of PV^+^ RGCs. OODS cells respond to both light onset and offset (ON-OFF), ON tr Sm cells respond primarily to light onset (ON), and OFF tr Me and OFF tr Sm cells respond primarily to light offset (OFF). An unidentified RGC Type 5 had mainly ON visual responses. A bright spot 225 μm in diameter centered on the soma was used as the stimulus (schematic on the left). The gray shading indicates pulse duration. Scale bars apply to all cells. (B) Representative responses of the five PV^+^ RGC subtypes to somatic current injections at different amplitudes (overlaid). Blueish colors indicate hyperpolarizing pulses; reddish colors indicate depolarizing pulses. The gray shading indicates pulse duration. Scale bars and color scheme on top right are the same for all cells. (C) Firing rate over time for the traces shown in (C). Same color scheme as in (C). (D) Representative tracings of four PV^+^ RGC subtypes, illustrating their dendritic architectures.

In a first set of experiments, we sought to examine responses of PV^+^ RGCs during somatic current injections, allowing us to study the intrinsic electrical properties of different types of RGCs in contrast to their well-studied responses during visual stimulation. We specifically targeted medium-sized (∼10–15 μm) PV^+^ RGCs, however, it is likely that smaller soma RGCs were also occasionally recorded. We did not aim to record from α RGCs as their intrinsic electrical properties have been studied in detail in a recent study by our group^21^; this was guaranteed by recording cells with somas smaller than 15 μm as well as testing for characteristic α RGC light responses.^21^ A simple light stimulation protocol was used to broadly classify RGC subtypes based on their spiking responses. 60–70% of cells responded to visual stimulation, allowing us to distinguish merely three RGC subclasses (ON, OFF, ON-OFF), although previous studies suggested at least 5 PV^+^ non-α RGC types.^22^ To achieve a more comprehensive classification, we therefore performed additional experiments involving somatic current injections, which enabled the identification of several subtypes. We investigated 85 cells, of which 71 exhibited light responses or responses to current injections that allowed us to categorize cells into 5 subtypes (Figure 1; Table 1). For the remaining 14 cells, we observed heterogeneous response features that could not be assigned to unique cell subtypes. Among the five classified subtypes, one responded to both the onset of bright and dark spot stimulation (ON-OFF), two were ON-type cells responding to the onset of bright spot stimulation, and two were OFF-type cells activated by dark spot stimulation (Figure 1A). As a next step, current was injected into the soma of RGCs to examine transient and sustained firing properties as well as action potential kinetics (Figures 1B and 1C). The identified subtypes were visually distinguishable during the experiment based solely on their responses to light stimulation (if available) and current injections (referred to as “experimenter-based” classification).

**Table 1:** Mapping of recorded PV^+^ RGCs to previously defined cell types

	Farrow et al.^22^	Bae et al.^3^	Goetz et al.^4^	Visual response	Confidence
Type 1	PV0	37c, 37d, 37r, 37v	**OODS** (D, T, V, N)	ON-OFF (33%)	high
Type 2	PV2	6sn	**ON tr Sm**	ON transient (100%)	high
Type 3	PV4	4on	**OFF tr Me**	OFF transient (86%)	medium-high
Type 4	PV4	4i	**OFF tr Sm**	OFF transient (82%)	medium-high
Type 5	?	?	?	ON (50%)	–

Five types of PV^+^ RGCs were mapped to previously established cell types introduced by Farrow et al.^22^, Bae et al.^3^ and Goetz et al.^4^ based on visual responses, intrinsic electrical responses as well as anatomical data. Numbers in parentheses indicate the percentage of cells responsive to visual stimulation.

**Figure 2:**
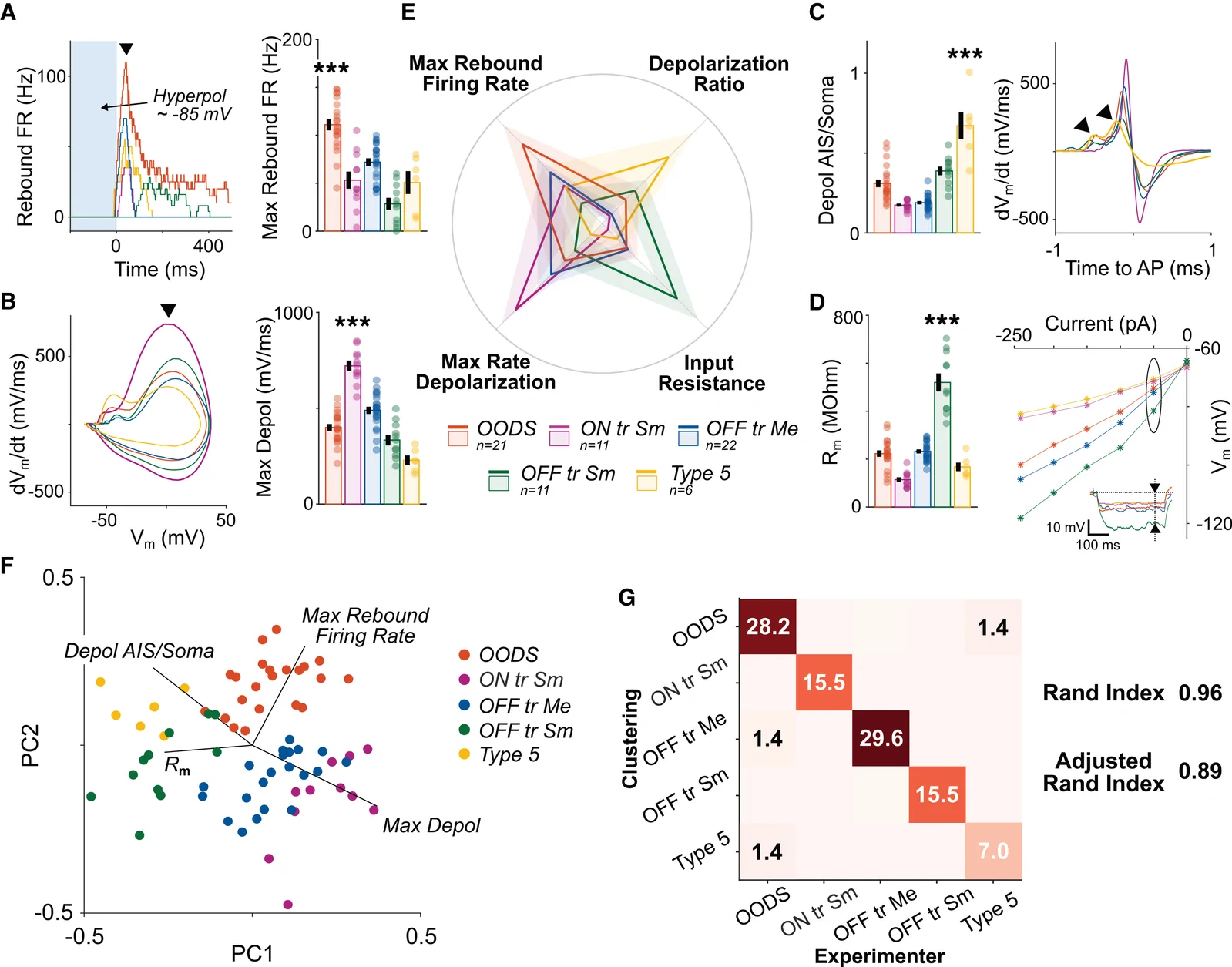
Distinct intrinsic electrical profiles define five PV^+^ RGC subtypes (A, left) Rebound firing rate over time in each PV + RGC type arising after current injections hyperpolarizing V_m_ to ∼ -85 mV (A, right) Comparison of maximum rebound firing rates (indicated by an arrowhead in the left panel) between the five PV^+^ RGC types. (B, left) The temporal derivative of V_m_ is plotted versus V_m_ (phase plot) for five representative PV^+^ RGCs. (B, right) Comparison of maximum rate of depolarization (indicated by arrowhead in left panel) between the five PV^+^ RGC types. (C, right) The temporal derivative of V_m_ is plotted over time in five representative PV^+^ RGCs. (C, left) Comparison of the ratio of AIS/soma maximum rate of depolarization (indicated by arrowheads in the right panel) between the five PV^+^ RGC types. (D, right) Voltage-to-current relationship for representative cells of the five PV^+^ RGC types. The inset shows raw traces for hyperpolarizing current injections with an amplitude of 50 pA (black ellipse in main panel). Arrowheads indicate the deflection of V_m_ to compute input resistance. (D, left) Comparison of input resistance between the five PV^+^ RGC types. Asterisks in A-D indicate a statistically significant difference for a given RGC type versus the four other types (one-way ANOVA + Tukey’s HSD, ∗∗∗*p* < 0.001). (E) Radar plot illustrating four electrophysiological parameters that broadly characterize PV^+^ RGC subtypes. Each direction represents one parameter, highlighting the distinct electrophysiological signature of four out of five PV^+^ RGC subtypes. Error bars and shadings in A-E indicate mean ± SEM. (F) PCA-based k-means clustering of PV^+^ RGC subtypes based on 19 electrophysiological parameters. The magnitude and sign of the contribution of the four parameters in A-D are shown. (G) Confusion matrix illustrating the clustering performance of the five PV^+^ RGC subtypes. The matrix displays the percentage of cells correctly and incorrectly assigned to each subtype based on electrophysiological parameters. Diagonal values represent the proportion of accurately classified cells, while off-diagonal values indicate misclassifications.

**Figure 3:**
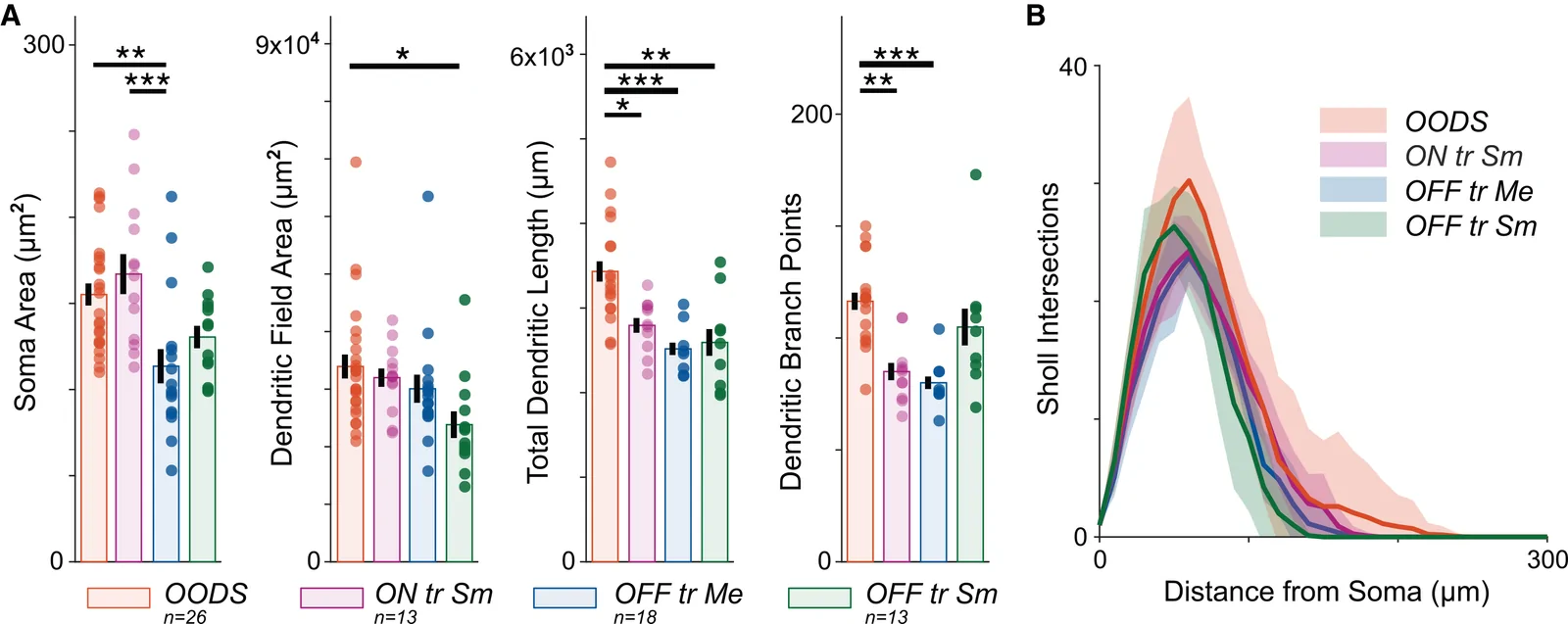
Somatic and dendritic diversity of PV^+^ RGC subtypes (A) Quantitative comparison of morphological parameters presented as bar plots, including soma area, dendritic field area, total dendritic length, and number of dendritic branch points (one-way ANOVA + Tukey’s HSD, ∗*p* < 0.05, ∗∗*p* < 0.01, and ∗∗∗*p* < 0.001). (B) Sholl analysis profiles of the four PV^+^ RGC subtypes, showing dendritic complexity as a function of radial distance from the soma. Data are represented as mean ± SEM in both panels.

**Figure 4:**
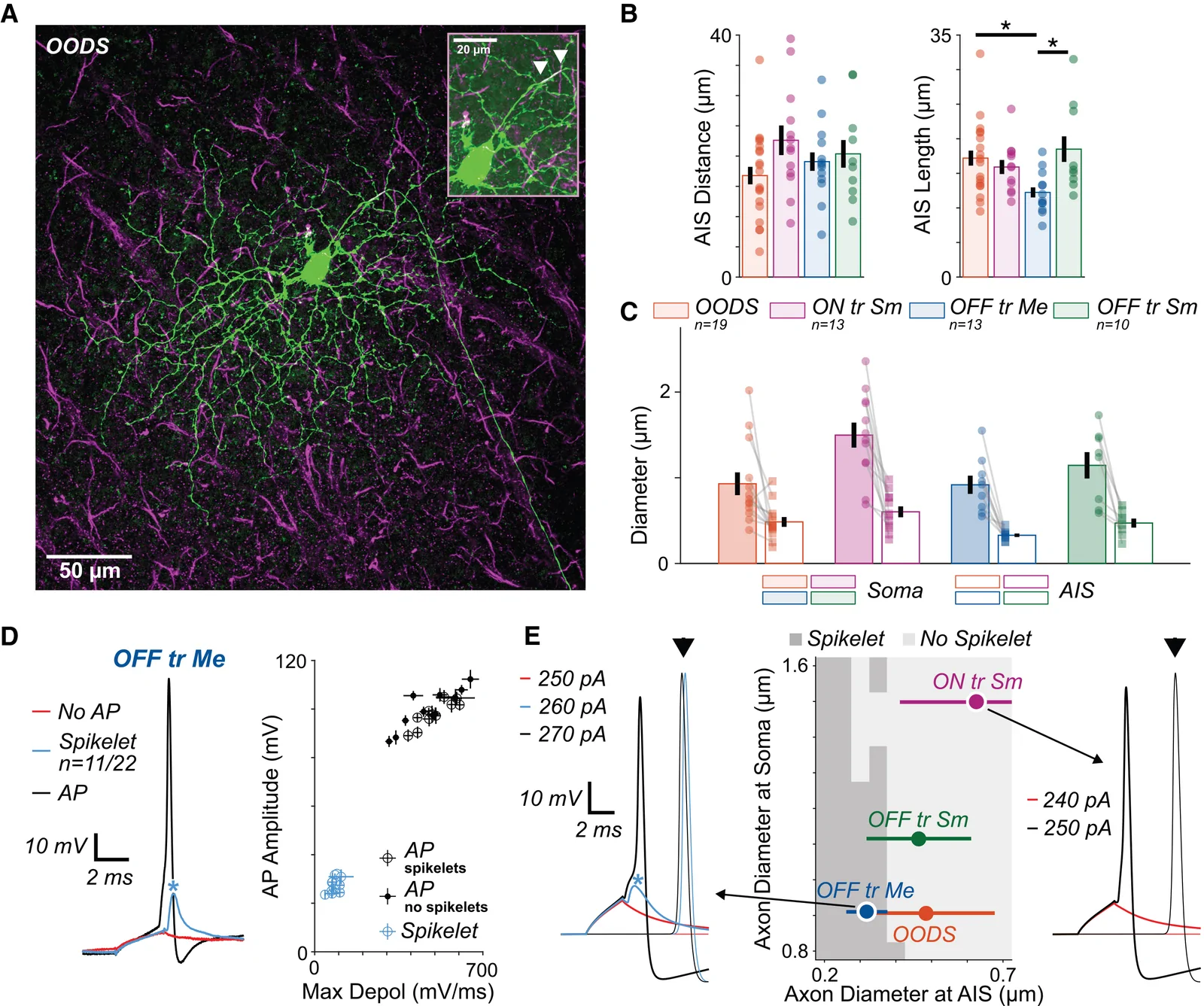
Proximal axon morphology impacts action potential backpropagation in PV^+^ RGCs (A) Confocal image of a Neurobiotin-filled OODS RGC labeled with β-spectrin IV to visualize the AIS. The inset shows an enlarged view of the soma and AIS (start and end marked by arrowheads). (B) Comparison of AIS distance from the soma and AIS length across four PV^+^ RGC subtypes (One-way ANOVA + Tukey’s HSD, ∗*p* < 0.05). (C) Comparison of diameters at the axon hillock versus at the AIS. Gray lines indicate measurements from the same cell. Data are represented as mean ± SEM in B and C. (D, left) Representative traces of axonally backpropagated action potentials in the soma in an OFF tr Me cell. In 50% of OFF tr Me RGCs, backpropagation failed when the stimulus was close to threshold, which was associated with distinct spikelets recorded at the soma (blue asterisk). (D, right) Peak amplitude versus maximum rate of depolarization is plotted for spikelets (blue) and full action potentials (black). (E) Modeled responses of action potential backpropagation in OFF tr Me (left) and ON tr Sm (right) RGCs. An elicited spikelet is indicated by the blue asterisk. Responses recorded at the distal axon are indicated by arrowheads. Spikelets were observed in cells with axon diameter configurations indicated in dark gray in the colormap (center). Measured axon diameters at the AIS (mean ± STD) from B are indicated for the four PV^+^ RGC types.

**Figure 5:**
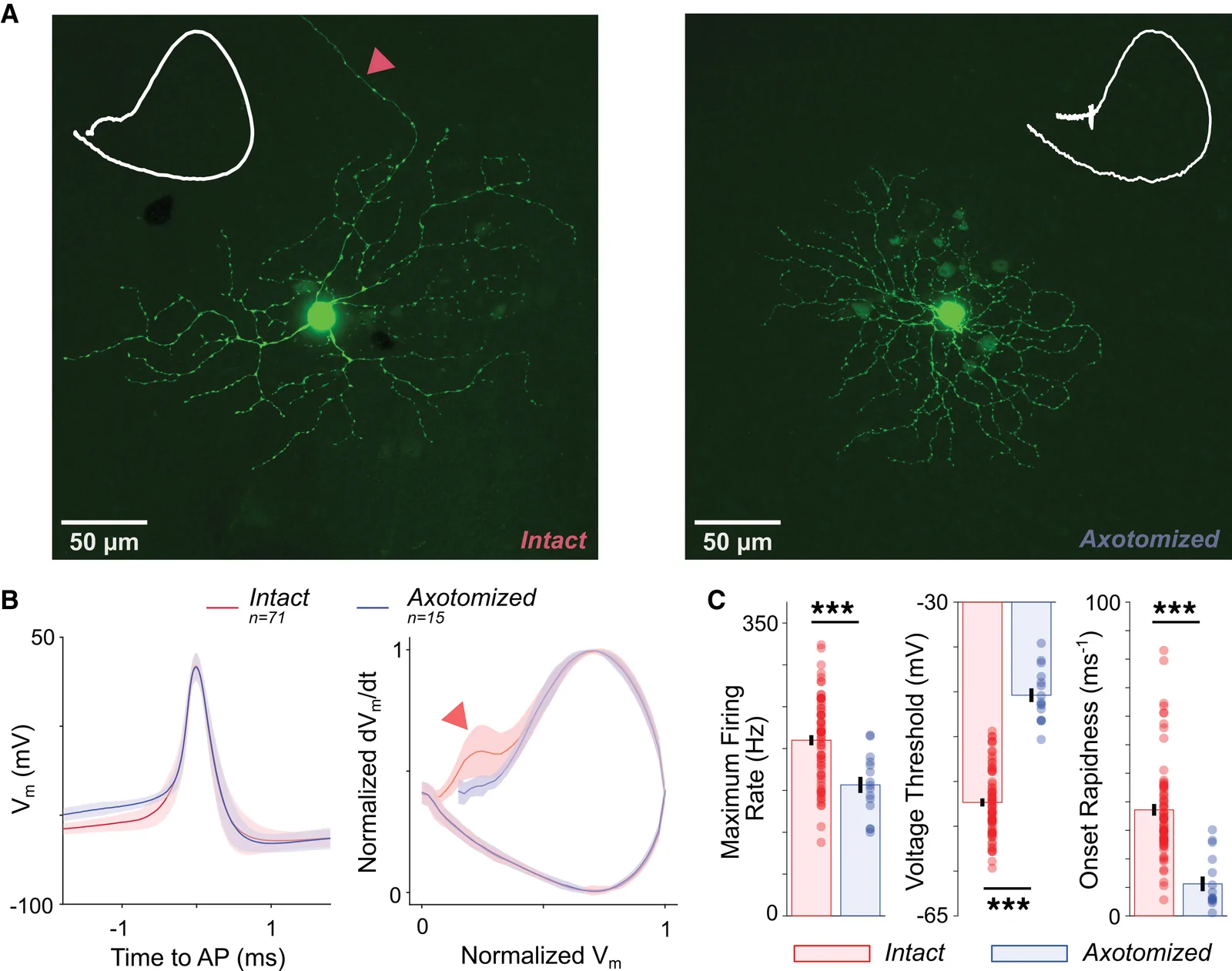
Axon-dependent electrophysiological signature is associated with impaired spike initiation and reduced excitability (A) Confocal images of two neurobiotin-filled PV^+^ RGCs. Left: ON tr Sm RGC with intact axon (indicated by red arrowhead). Right: OFF tr Sm RGC lacking an axon (axotomized). Phase plots shown in the upper corners were derived from electrophysiological recordings of the same cells. (B, left) Electrophysiological comparison of intact (red) and axotomized (blue) unfilled PV^+^ RGCs. V_m_ over time illustrating differences in spike initiation. (B, right) Normalized phase plot (dV_m_/dt versus V_m_) for RGCs with and without axons. The red arrowhead indicates the distinct “hump” associated with axonal action potential initiation. (C) Quantitative comparison of intact versus axotomized unfilled PV^+^ RGCs. The three bar plots show maximum firing rate (left, *t* test), voltage threshold (middle, *t* test), and onset rapidness (right, Wilcoxon rank-sum test) for intact (red) and axotomized (blue) cells. Data are represented as mean ± SEM in B and C, ∗∗∗*p* < 0.001.

During current injection experiments, a subset of cells was filled with Neurobiotin, and subsequently cells were immunostained and reconstructed, enabling anatomical tracing (Figure 1D). Analysis of the recorded data revealed significant differences in action potential kinetics and other spiking-related parameters between Neurobiotin-filled and unfilled cells (Figure S1;^23^). Therefore, data from unfilled cells were used to further analyze intrinsic electrical properties (Figures 1A–1C, 2, 5B–5C), whereas filled cells were used for anatomical characterization including AIS measurements (Figures 1D, 3, 4, and 5A, see Methods).

As summarized in Table 1, our Type 1 RGC likely corresponds to the PV0 subtype in Farrow et al.,^22^ which aligns with the ON-OFF direction-selective RGCs (OODS D, T, V, N RGCs) described by Goetz et al.^4^ This correspondence is supported by shared morphological and functional characteristics (see later in discussion for details): the Type 1 RGC correspond to the PV0 subtype as both are ON-OFF types and bistratified, and exhibit dendritic fields of comparable size, ranging from approximately 20,000–70,000 μm^2^. Notably, in Farrow et al.,^22^ the PV0 subtype was the only cell type exhibiting a robust ON-OFF light response; similarly, in Goetz et al.,^4^ OODS cells represented the only ON-OFF population with dendritic field sizes matching those of our Type 1 RGCs. Our Type 2 RGC corresponds to the PV2 cell type in Farrow et al.^22^ and was matched to the ON transient small receptive field (ON tr SmRF) RGC described by Goetz et al.^4^ This type represents the only PV^+^ non-α ON-type RGC with a high firing rate in Farrow’s classification, consistent with our Type 2 population. In addition, this subtype is also characterized by high firing rates: Goetz et al.^4^ reported peak firing rates of ∼350 Hz for ON tr SmRF RGCs, which closely matches our observations, where most Type 2 RGCs exhibited peak firing rates in the ∼250–330 Hz range. Further support for this classification comes from cross-study comparisons. Chang et al.^24^ identified their tOn-small RGC subtype as corresponding to both PV2 and ON tr SmRF RGCs. Notably, the tOn-small subtype shares key features with our Type 2 cells, including an ON-type transient light response as well as comparable soma (∼150 μm^2^) and dendritic field area (∼31,000 μm^2^). Types 3 and 4 RGCs correspond to the PV4 type in Farrow et al.,^22^ as both are OFF-type cells with relatively similar dendritic field areas (approx. ∼20,000–30,000 μm^2^). However, since Farrow et al.^22^ did not perform measurements of intrinsic electrical properties, these two subtypes probably were not further distinguished as they have similar OFF transient light responses (Figure 1A). Based on our data, we identified Type 3 as corresponding to the OFF tr MeRF RGC described by Goetz et al.,^4^ as it exhibits a larger dendritic field area compared to Type 4 RGC (Type 3: ∼30,000 μm^2^ vs. Type 4: ∼24,000 μm^2^). Accordingly, Type 4 corresponds to the OFF tr SmRF subtype. Finally, Type 5 represents a tentative ON-type RGC that we were unable to conclusively identify due to the lack of morphological reconstructions.

### Electrophysiological properties define five PV^+^ RGC subtypes

In a next step, we checked the reliability of the experimenter-based clustering approach by performing an unlabeled clustering method. First, we analyzed the spiking responses of recorded RGCs in detail during intracellular stimulation with short (3 ms) and long (500 ms) current injections. Based on these observations, we found single electrophysiological parameters that, on average, characterized four out of the five subtypes: OODS RGCs exhibited the highest rebound firing rate following hyperpolarization (110 ± 28 Hz, Figure 2A), ON tr SmRF RGCs were characterized by the highest rate of depolarization (721 ± 90 mV/ms, Figure 2B), OFF tr SmRF RGCs showed the largest input resistance (520 ± 124 MΩ, Figure 2D), and the unmatched RGC Type 5 displayed the highest AIS/Soma depolarization rate ratio (0.67 ± 0.20, Figure 2C). In contrast, we did not find a single electrophysiological parameter that could serve as a clear distinguishing feature of OFF tr MeRF cells; however, their characteristic response to hyperpolarizing pulses with little sag as well as a short and strong hyperpolarization-induced rebound (Figures 1B and 1C) were reliable indicators for this cell type. A radar plot visualizes the five PV^+^ subtypes and their distinct electrophysiological responses (Figure 2E).

To obtain a more robust classification, we analyzed the dataset by principal component analysis (PCA) followed by k-means clustering (Figure 2F). We used the 19 non-redundant parameters from short and long current injection experiments. The clustering approach shown in Figure 2F indicates how the four parameters shown in Figures 2A–2D delineate broad clusters in a 2D representation of the first two principal components. To validate how well the experimenter-based assignment from light-evoked responses and visual inspection matched the multidimensional clustering, we computed a confusion matrix indicating that only 3 out of 71 cells were misclassified (Figure 2G). The high classification accuracy (Rand Index 0.96; Adjusted Rand Index 0.86) confirmed subtype-specific intrinsic response characteristics in each of the five PV^+^ RGCs.

### Somatic and dendritic diversity of PV^+^ RGC subtypes

In addition to electrophysiological classification, we filled a subset of PV^+^ RGCs with Neurobiotin to allow for morphological reconstruction and tracing. We characterized PV^+^ RGC subtypes anatomically to obtain a more comprehensive view of their properties. As we infrequently found Type 5 during the experiments, we were not able to reconstruct cells for morphological analysis. The remaining four subtypes (OODS, ON tr SmRF, OFF tr MeRF, and OFF SmRF) are analyzed later in discussion. Representative tracings of these four PV^+^ RGC subtypes are shown in Figure 1D. OODS RGCs were the only ones among the detected subtypes that were bistratified, making the classification of this cell type unambiguous. The other cell types were classified by a combination of light responses and intrinsic electrical properties (“experimenter-based”).

Quantitative comparisons are presented in Figure 3A. We measured soma area and dendritic field area across 70 filled RGCs. Total dendritic length and dendritic branch points were extracted from 45 reconstructed tracings. The results revealed that the OFF tr MeRF cells exhibited the smallest soma area, followed by OFF tr SmRF with an intermediate soma size, whereas OODS and ON tr SmRF displayed the largest soma areas. In contrast to these soma-related parameters, OFF tr SmRF showed the smallest dendritic field area, while the other three cell types possessed dendritic fields of approximately equal size. When examining the total dendritic length across the different cell types, OODS emerges as significantly longer than the remaining three subtypes. This may be explained by the fact that OODS cells are bistratified, essentially owning two distinct dendritic trees. This observation is further reflected in the number of dendritic branching points, where OODS again demonstrates the highest degree of branching compared to the other three cell types. Notably, the OFF tr SmRF subtype, despite exhibiting the smallest dendritic field area and a relatively low total dendritic length, represents the second most highly branched cell type.

To further capture differences in dendritic architecture, we performed Sholl analysis of confocal image reconstructions (Figure 3B). The Sholl plots demonstrated that ON tr SmRF and OFF tr MeRF had the fewest peak intersections, while OODS cells displayed the highest number of peak intersections overall. Additionally, OFF tr SmRF cells exhibited denser branching near the soma, leading to a steep increase in intersections, followed by a faster decrease with distance compared to the other subtypes. This proximal branching contributed to the overall more complex architecture of OFF tr SmRF RGCs.

### Distinct AIS features and axon diameters affect PV^+^ RGC physiology

The AIS is a sodium channel-rich compartment that initiates action potentials and ensures their reliable signal propagation in the central nervous system. Once generated in the AIS, action potentials propagate forward into the axon to drive synaptic transmission but also backpropagate into the soma. The AIS has to depolarize the large somatodendritic compartment by generating an axial current that brings the soma to threshold, and subsequently, somatic sodium channels regenerate the spike. The strength of this current depends primarily on proximal axon geometry and sodium channel density rather than AIS length.^25^ To determine whether these structural properties influence function, we next analyzed the impact of AIS length, location, and proximal axon geometry, i.e., diameter at the soma and at the proximal AIS, on action potential backpropagation in PV^+^ RGCs.

Figure 4A shows a confocal image of an OODS RGC (green), with the AIS identified by β-spectrin IV immunostaining (magenta). Across all five subtypes, AISs were clearly delineated as discrete proximal axonal segments on average 15–20 μm distant from the soma. Quantification revealed no significant subtype differences in AIS distance from the soma, but AIS length varied considerably (Figure 4B). OFF tr MeRF cells exhibited, on average, the shortest AISs (12.2 ± 3.1 μm) in comparison to the other subtypes. We next examined proximal axon geometry by comparing diameters at the soma and at the AIS origin (Figure 4C). Among the RGCs, OFF tr MeRF RGCs had, on average, the smallest axon diameters, both at the soma and the AIS (soma: 0.92 ± 0.32 μm, AIS: 0.33 ± 0.06 μm), while ON tr SmRF RGCs exhibited the largest axon diameters at both areas (soma: 1.50 ± 0.51 μm, AIS: 0.60 ± 0.22 μm). Consistent with the report of Raghuram et al.^26^ in α RGCs, we also observed that the axon diameter tapered between these compartments in all PV^+^ subtypes, although the degree of narrowing varied, suggesting subtype-specific structural tuning of axial current flow.

Electrophysiological recordings of OFF tr MeRF RGCs revealed specific responses to short current injections close to threshold. In 50% of OFF tr MeRF RGCs, action potentials elicited near threshold failed to successfully backpropagate to the soma. Instead, small-amplitude somatic spikelets were detected (Figure 4D, left), characterized by reduced peak amplitude and slower depolarization kinetics relative to full action potentials (Figure 4D, right). Such spikelets were absent in other PV^+^ RGC subtypes and were not reported in previous α RGC studies.^13^ Action potential kinetics in OFF tr MeRF RGCs that fired spikelets were similar to action potential kinetics in cells without spikelet generation, indicating normal somatic action potential generation combined with an impaired backpropagation of the AIS-initiated spike into the soma (Figure 4D, right).

To probe proximal axon geometry as a potential underlying cause for spikelet propensity, we investigated the effect of proximal axon diameter in a computational model of action potential generation (Figure 4E). Whereas modeled spiking responses were relatively unaffected by the axon diameter at the soma (Figure 4E, right), model neurons with a thin axon diameter at the AIS were prone to fire spikelets similar to recorded OFF tr MeRF RGCs (Figure 4E, left). The identified parameter space in which proximal AIS narrowing favored spikelet generation is indicated in dark gray in Figure 4E. The model also allowed us to record spiking activity in the distal axon (Figure 4E, left, arrowheads), which demonstrates unidirectional signal propagation into the axon associated with failed backpropagation (spikelet) at stimuli close to threshold for small AIS diameter cells. Notably, axon diameter measurements at the proximal AIS in OFF tr MeRF RGCs from C aligned with the spikelet-prone zone, whereas OODS, ON tr SmRF, and OFF tr SmRF fell outside of it.

Together, these results demonstrate that PV^+^ RGC subtypes differ not only in AIS length but also in proximal axon narrowing, both of which critically shape the axial current that supports action potential backpropagation. The unusually sharper narrowing in OFF tr MeRF RGCs together with shorter AISs may underlie their propensity for spikelet generation, revealing subtype-specific action potential backpropagation. However, other factors such as sodium channel density and type can also play a role in action potential backpropagation.^27^^,^^28^

### Functional differences between intact and axotomized PV^+^ RGCs

Since the AIS is known to play a critical role in action potential initiation and propagation,^29^ we next compared recordings from PV^+^ RGCs with intact axons to those in which the axon was inadvertently severed during opening of the inner limiting membrane to access RGC somata for patch-clamp recordings. Investigating PV^+^ RGCs lacking an axon is particularly valuable, as it may provide insights relevant to conditions in which neurons sustain axonal damage or fail to develop an AIS.

Figure 5A illustrates this comparison, showing confocal images of an ON tr SmRF RGC with an intact axon and an OFF tr SmRF RGC lacking an axon. For each example, the corresponding phase plot derived from electrophysiological recordings is shown in the corner. Notably, in the absence of an AIS, the initial rising component of the phase plot was flattened, indicating impaired spike initiation. We also noticed that neurons filled with Neurobiotin exhibited altered phase plots compared to unfilled cells. Specifically, the hyperpolarizing component of the phase plot appeared markedly flattened. This difference is illustrated in Figure S1A, with OODS RGCs displaying a clear shift in the rate of de- and hyperpolarization. Moreover, action potential duration was increased in Neurobiotin-filled cells, suggesting that the dye significantly altered action potential properties (Figure S1B). For the reasons described above, we did not use Neurobiotin-filled cells for electrophysiological analyses but defined axotomized cells as unfilled cells lacking the hump in the phase plot. As shown in Figure 5B, axotomized PV^+^ RGCs required stronger depolarization to reach action potential threshold, reflecting the changes in their phase plot (red arrowhead). Quantitative analyses confirmed these effects (Figure 5C). Compared with intact cells, axotomized PV^+^ RGCs exhibited a reduced maximum firing rate and slower onset rapidness, both indicative of decreased excitability. In addition, the voltage threshold was significantly elevated by approximately 12 mV in axotomized cells compared with intact cells (−40.4 ± 3.0 vs. −52.3 ± 3.8 mV), highlighting the role of the AIS for low-threshold spike initiation in RGCs. These results demonstrate that the AIS is critical for maintaining normal excitability and efficient spike initiation in PV^+^ RGCs. While we interpret the observed changes in excitability and firing properties primarily in the context of AIS disruption, we cannot exclude that other, injury-related effects might also play a role. However, recorded cells lacking an AIS showed brisk spiking responses, no shift in baseline activity, and could be recorded for extended periods of time.

## Discussion

As shown in this study, medium-sized soma PV^+^ RGCs are highly diverse in their intrinsic electrical and anatomical properties and constitute a heterogeneous population beyond the large-sized soma PV^+^ RGCs. Nevertheless, we identified five PV^+^ RGC subtypes that can be clearly distinguished from one another based on their light responses as well as their intrinsic electrical properties and anatomical parameters. Moreover, we observed additional diversity depending on whether these cells possess an axon including an AIS or not, and the diameter of the axon (at the soma vs. at the AIS) also proved to be subtype-specific.

### Electrical and anatomical diversity in PV^+^ RGC subtypes

In our previous publication,^13^ we detailed the anatomical and electrophysiological properties of α RGCs. In the present study, we extended this approach to medium-sized soma PV^+^ RGCs. The five identified medium-size soma PV^+^ RGC subtypes could be unambiguously distinguished by a combination of three complementary approaches: their responses to light stimulation, their responses to current injections, and their anatomical features (Figures 1, 2, 3, and 4). Importantly, the experimenter-based classification (based on light and current injection responses during the experiment) proved to be reliable, with approximately 96% of cells correctly assigned to their respective subtype, which was confirmed by PCA followed by k-means clustering. The majority of misclassifications occurred among the unmatched subtype Type 5, which was occasionally assigned to either OFF tr MeRF or OODS based solely on their intrinsic electrical properties. Anatomical analyses provided additional discriminative power; for example, we found that OODS RGCs exhibited a bistratified dendritic morphology, resembling the PV0 cells described by Farrow et al.,^22^ who also used a PV^+^ mouse line. We also found that ON tr SmRF RGCs correspond to Farrow’s PV2 cells, as both are ON-type cells characterized by small-medium dendritic field areas and the highest firing rates. Furthermore, the tOn-small RGC described in Chang et al.^24^ likely corresponds to the ON tr SmRF cells, since it is an ON-type transient subtype and exhibits similar soma and dendritic field areas. OFF tr MeRF and OFF tr SmRF RGCs are likely to correspond to Farrow’s PV4 type, as both types exhibit OFF-transient responses to visual stimulation; however, their intrinsic electrical properties show significant differences (Figures 1 and 2). Since Farrow et al.^22^ did not perform somatic current injections revealing intrinsic electrical properties, these features remained unidentified in their study. The unmatched subtype Type 5 could not be matched to another type in the recent classification scheme due to missing anatomical data. Importantly, we did not match any of our cells to Farrow’s PV7 type, which was characterized by a small dendritic field area (∼10,000 μm^2^) and an asymmetric dendritic tree.^22^ Most likely, our targeting approach of cells with somas larger than 10 μm was the reason for not recording from this cell type.

In our experiments, we used a 225 μm spot and a single contrast step, which is not optimized to effectively stimulate small-medium-sized RGCs. This limits the discriminative power of RGCs based solely on their light responses in our study. In the future, the identification process could be optimized by using a light stimulus protocol similar to the one used by Goetz et al.^4^ in combination with somatic current injections.

### Axonal specializations shape PV^+^ RGC physiology

The AIS is a critical site for action potential initiation, and its structural and molecular organization profoundly influences the excitability of RGCs.^29^^,^^30^ Voltage-gated sodium (Nav) channels, particularly Nav1.2 (proximal) and Nav1.6 (distal),^27^ are clustered at the AIS through interactions with the scaffold protein ankyrin G (ankG) and the actin-β-spectrin IV cytoskeleton.^17^ The AIS is not only involved in action potential propagation, but is also a part of the neuron, which is highly organized to maintain network homeostasis,^31^ therefore, it can undergo dynamic reorganization in response to stimulation, meaning elongation/shortening/relocating^32^^,^^33^^,^^34^ for a longer time period or ion channel adaptation for rapid responses.^35^^,^^36^

In this study, we demonstrate that AIS length of PV^+^ RGC subtypes differs significantly from one another, with OFF tr MeFR exhibiting the shortest AISs (Figure 4). In contrast, the AIS distance from the soma did not differ significantly among the four investigated subtypes. We further found that OFF tr MeFR RGCs possess the thinnest axons, and that OFF tr MeFR RGCs uniquely display spikelets near threshold in 50% of cases (Figure 4). Consistent with these findings, our computational modeling showed that only the modeled OFF tr MeFR RGCs generated spikelets, whereas the other subtypes (OODS, ON tr SmRF, OFF tr SmRF) did not, suggesting that subtype-specific axonal geometry modulates current flow and excitability. We also performed patch-clamp recordings and intracellular fills of RGCs with and without an AIS (Figure 5). Quantitative analysis revealed that the loss of the AIS leads to pronounced changes in excitability and spike initiation in PV^+^ RGCs, similar to what was shown previously in α RGCs^14^ as well as in Nav1.6 knockout mice in neocortical pyramidal neurons.^37^ Furthermore, computational modeling suggests similar axonal AP initiation characteristics when AIS sodium channel density is substantially lowered, as presented here for axotomized cells.^28^ Axotomized PV^+^ RGCs exhibited reduced maximal firing rates and slower onset kinetics, as well as elevated voltage thresholds, highlighting the importance of an intact axon for functional output of PV^+^ RGCs. Raghuram et al.^26^ investigated how AIS geometry scales with overall cell size in ON α sustained RGCs and demonstrated that both AIS length and distance from the soma increase proportionally with soma size. Using computational modeling, they further showed that reducing AIS length by 50% leads to a marked decrease in onset rapidness, highlighting the sensitivity of spike initiation dynamics to AIS morphology. This relationship closely parallels our observations in axotomized PV^+^ RGCs, which also exhibited diminished onset rapidness, suggesting that AIS shortening or loss may underlie similar excitability deficits. Furthermore, Werginz et al.^14^ demonstrated that mechanical removal of the AIS in dorsal OFF-α RGCs abolished their sustained firing behavior, causing them to display ventral-like transient response patterns. This finding indicates that the AIS is essential for maintaining the intrinsic excitability properties of RGCs, and that its absence leads to profound alterations in spike generation dynamics. Wienbar et al.^15^ showed that the intrinsic properties indeed affect the output signals, rather than the synaptic inputs in the mouse. They compared bSbC and OFF sustained α RGCs and found that the low level of Nav1.6 ion channels, shorter AIS, and low sodium conductance in bSbC lead to depolarization block in response to negative contrast stimulus. In summary, our results here and results from other recently published studies suggest substantial effects of the AIS on action potential initiation as well as backpropagation in RGCs.

### Limitations of the study

In our experiments, we applied a 225 μm light spot and a single contrast-step stimulus, conditions that are not ideally suited for activating small-to medium-sized RGC subtypes. As a result, classification based exclusively on light-evoked responses was limited in resolution. Future studies could improve subtype identification by implementing a more refined visual stimulation paradigm, similar to that described by Goetz et al.,^4^ combined with somatic current injection protocols.

## Resource availability

### Lead contact

Requests for further information and resources should be directed to and will be fulfilled by the lead contact, Paul Werginz (paul.werginz@tuwien.ac.at).

### Materials availability

This study did not generate new unique reagents.

### Data and code availability

#### Data

All data reported in this paper will be shared by the lead contact upon request.

#### Code

All code used in this study has been deposited at TU Wien Research Data Repository and is publicly available at https://doi.org/10.48436/jmf7t-tek93 as of the date of publication.

#### Additional information

Any additional information required to reanalyze the data reported in this paper is available from the lead contact upon request.

## Acknowledgments

We would like to thank Jae-Ik Lee and Andrea Corna for providing feedback and comments on the manuscript and Mai Thu Bui for excellent technical assistance. We thank Maren Engelhard for the β-spectrin IV antibody. This research was funded in whole or in part by the Austrian Science Fund (10.13039/501100002428FWF) [10.55776/P35488]. For open access purposes, the author has applied a CC BY public copyright license to any author-accepted manuscript version arising from this submission.

## Author contributions

V.K. and P.W. conceived the study. V.K. and P.W. performed the electrophysiology experiments. V.K. performed the immunochemistry and confocal imaging experiments as well as tracings. P.W. performed computational modeling. V.K., G.Z., and P.W. analyzed the results, wrote the manuscript, and prepared the figures.

## Declaration of interests

The authors declare no competing interests.

## STAR★Methods

### Key resources table

**Table:** 

REAGENT or RESOURCE	SOURCE	IDENTIFIER
**Antibodies**		

βIV-Spectrin (amino acids 2237–2256 of human ßIV-spectrin)	Maren Engelhardt, JKU, Linz, Austria^39^	–
Donkey anti-Rabbit IgG (H + L) Alexa Flour Plus 647	Thermo Fisher Scientific	Cat#A32795; RRID: AB_2762835
Neurobiotin **®** Tracer	Vector Laboratories	Cat#SP-1120; RRID: AB_2313575
Streptavidin, Alexa Fluor™ 405 conjugate	Thermo Fisher Scientific	Cat#S32351

**Experimental models: organisms/strains**		

Ai32: ChR2H134R-EYFP driven by Pvalb-IRES-Cre	The Jackson Laboratories	crossed from B6.129P2-Pvalb^tm1(cre)Arbr^/J, Stock Number: 017320 RRID:IMSR_JAX:017320 and B6.Cg-*Gt(ROSA)26Sor*^*tm32(CAG-COP4*^*∗*^*H134R/EYFP)Hze*^/J, Stock Number: 024109 RRID:IMSR_JAX:024109

**Software and algorithms**		

MATLAB	Mathworks	Version R2022b
TREES Toolbox (MATLAB)	https://www.treestoolbox.org^40^	Version 1.15
LAS X Office	Leica	Version 1.4.9.30653
ImageJ/FIJI	NIH	Version 1.54g
PatchMaster Next	HEKA	Version 1.4.1
GEARS	https://www.gears.vision^41^	Version 0.4
NEURON	https://www.neuronsimulator.org^45^	Version 8.2
Python	https://www.python.org	Version 3.13
Custom written Python + NEURON code	–	https://doi.org/10.48436/jmf7t-tek93

### Experimental model and study participant details

For electrophysiology experiments, we used the mouse reporter line Ai32: ChR2H134R-EYFP driven by Pvalb-IRES-Cre, which was crossed from B6.129P2-Pvalb^tm1(cre)Arbr^/J, Stock Number: 017320 and B6.Cg-*Gt(ROSA)26Sor*^*tm32(CAG-COP4*^*∗*^*H134R/EYFP)Hze*^/J, Stock Number: 024109).^42^ Mice were kept and handled at the Medical University of Vienna under the approval of the Austrian Federal Ministry of Science and Research in accordance with the Austrian and EU animal laws (BMWFW-66.009/0403-WF/V/3b/2014). Adult (>P30) male and female mice were used in this study see Table below, potential sex-specific differences were not investigated.

**Table:** Number of recorded PV^+^ RGCs for electrophysiology and anatomical reconstructions

		OODS	On tr Sm	OFF tr Me	OFF tr Sm	Type 5	Total
Unfilled	*intact* ephys dataset	21 cells (17 retinas)	11 (9)	22 (13)	11 (11)	6 (6)	71 (35)
	*axotomized* ephys dataset	–	–	–	–	–	15 (12)
NB-filled	*intact* anatomy dataset	26 (26)	13 (13)	18 (18)	13 (13)	–	70 (70)

The intact electrophysiology dataset contains PV^+^ RGCs with an axon, whereas the axotomized electrophysiology dataset contains PV^+^ RGCs without an axon as inferred from phase plots of single action potentials. Data from the intact electrophysiology dataset were used for electrophysiological characterization and cell type identification (Figures 1A–1C, 2, 5B, and 5C). Data from the axotomized electrophysiology dataset were used for axon-dependent signature analyses (Figures 5B and 5C). The intact anatomy dataset contains Neurobiotin (NB)-filled PV^+^ RGCs with an axon. Data from the anatomy dataset were used for anatomical characterization (Figures 1D, 3, 4, and 5A). In total 80 retinas were used.

### Method details

#### Electrophysiology

Mice were sacrificed by cervical dislocation, eyeballs were extracted, and the lens was removed. The prepared eyeballs were kept in a transfer vial filled with oxygenated Ames medium (pH 7.4, Sigma-Aldrich or United States Biological) and brought to TU Wien. After cleaning from vitreous humor, the retina was mounted onto a filter paper in a Petri dish and placed under the microscope (40× magnification). The retina was perfused with oxygenated Ames medium at 33°C–35°C for the duration of the experiment.

For patch clamp experiments, we used an EPC10 USB amplifier controlled by PatchMaster Next (both HEKA); all recordings were obtained at a sampling rate of 100 kHz. Patch pipettes were filled with intracellular solution containing 125 mM K-gluconate, 10 mM KCl, 10 mM HEPES, and 10 mM EGTA (Carl Roth) and 4 mM Mg-ATP and 1 mM Na-GTP (Sigma-Aldrich). Pipettes were pulled with a micropipette puller (Sutter, P-1000) to obtain an optimal pipette resistance of 2–8 MΩ.

PV^+^ RGCs were targeted in the peripheral portion of the retina to avoid axon bundles close to the optic disc. RGCs were identified as PV^+^ cells, which express enhanced yellow fluorescent protein detectable under the fluorescence microscope,^20^ before opening the inner limiting membrane. Medium-size soma (diameter of ∼10–15 μm) cells were identified as PV^+^ RGCs in the RGC layer of the retina^21^^,^^43^ by epifluorescence microscopy. The duration of the targeting procedure was limited to a minimum (<1 s) and consisted of opening the shutter, marking fluorescent RGCs and closing the shutter. While during this process the retinal network including RGCs will be activated, a pause between the targeting procedure and actual recording (>5 min) ensured return to baseline activity levels in RGCs.

After targeting, a pipette was directed toward the RGC layer to break the inner limiting membrane and obtain access to the RGC soma. After that, another pipette was filled with intracellular solution to target the identified PV^+^ RGC. Following break-in, the series resistance was compensated with the bridge balance circuit of the amplifier. The liquid junction potential of −8 mV was corrected offline. All cells were held at a membrane voltage of approximately −65 mV with the low-frequency voltage clamp mode of the patch amplifier.

Two current injection protocols were used to probe intrinsic electrical properties: stimulus amplitudes of the long protocol (500 ms) ranged from -250-1000 pA; stimulus amplitudes of the short protocol (3 ms) ranged from 10 to 300 pA. The long protocol was used for spike train analysis, while the short protocol was used for threshold analysis. Light stimulation protocols were programmed in GEARS.^41^ Bright and dark spots (100% Weber contrast) with a diameter of 225 μm were projected onto the retina for 1 s through the 40× objective of the microscope, with a gray background of approximately 0.005 mW/mm^2^. Stimuli were repeated five times and breaks in between were 1.5 s long. While light levels were sufficient to activate photoreceptors, stimuli were well below the limit to drive optogenetic activity in RGCs (>0.5 mW/mm^2^,^42^).

#### Retina preparation for immunofluorescence staining and confocal imaging

For anatomical analysis, medium-size soma (∼10–15 μm in diameter) RGCs were filled with intracellular solution including 0.5% Neurobiotin tracer (Vector Laboratories, SP-1120). After finishing the experiment, the retina was fixed with 4% PFA (Invitrogen) for 30 min, then washed 3x for 15 min with 10X PBS (Invitrogen). For permeabilization, the retina was treated with 0.5% PBS-TX (10X PBS with 0.5% Triton X-100, Sigma-Aldrich) for 20 min at room temperature. Afterward, the retina was blocked with 3% BSA (Sigma-Aldrich) plus 0.5% TX for 4 h at 4°C. After removing the blocking solution, the retina was incubated in the primary antibody solution of 1% BSA +0.5% TX + β-Spectrin IV (rabbit, 1:1000) at 4°C overnight. The β-Spectrin IV primary antibody was provided by Maren Engelhardt (Johannes Kepler University Linz, Austria) and was published by Gutzmann et al.^39^ β-spectrin IV is a core structural component of the AIS cytoskeleton and is recruited to the AIS by ankyrin-G during AIS assembly.^44^ The next day, the retina was washed three times with 0.5% PBS-TX for 15 min. A reconstituted streptavidin Alexa Fluor 405 conjugate (Invitrogen) was 1:200, and the donkey anti-rabbit IgG (H + L) Alexa Fluor Plus 647 secondary antibody (ThermoFisher) 1:500 was diluted in 3% BSA plus 0.5% TX solution and added to the retina for overnight incubation at 4°C. The next day, the retina was washed three times with 0.5% PBS-TX for 15 min and fixed with 4% PFA for 10 min at room temperature. PFA was washed out 3× with 10X PBS twice for 15 min. The retina was finally mounted and coverslipped with a ProLong Glass Antifade Mountant (Invitrogen).

Stained retina samples were kept in a dark box at 4°C until they were imaged with the confocal microscope Leica Stellaris 5 (DMi8) at 40× magnification (HC PL APO 40×/1.10 W objective). Leica’s Lightning feature was used for the AIS imaging and Leica X LAS software was used to take and analyze the images. Tracing of the filled RGCs was made with the TREES toolbox^40^ in MATLAB (Mathworks). The images were analyzed based on findings from previous studies on α RGCs.^13^

#### Computational modeling

Multi-compartment models of RGCs followed methods described previously.^13^ Simulations were performed in NEURON 8.2^45^ controlled by Python 3.11. Models were based on simplified RGC morphologies with a spherical soma (diam = 12 μm), a simplified dendritic tree consisting of 4 main dendrites each 1000 μm in length with tapering diameter from 4 to 0.5 μm. The proximal axon (soma to AIS, L = 18 μm) was modeled as a tapered cylinder with diameters ranging between 0.8 and 1.6 μm at the soma and 0.2–0.7 μm at the AIS. The AIS (L = 13 μm) diameter tapered from a proximal diameter based on above values to 0.6 μm at its distal end. Ion channel dynamics were based on the RGC membrane model from Fohlmeister et al.^38^ A Nav1.6 channel located at the AIS was modeled by shifting the activation curves for m and h gates of the standard sodium channel by 6 mV (more hyperpolarized). Model temperature was set to 30°C similar to experimental conditions. Intracellular resistivity and specific membrane capacitance were set to 140 Ω∗cm and 1 μF/cm^2^, respectively. Ion channel densities can be found in Below Table.

**Table:** Ion channel densities along the neural membrane

	Dendrites	Soma	Soma-AIS	AIS	Axon
g_Na,1.2_	50	50	75	0	65
g_K,1.2_	48	48	48	0	40
g_Na,1.6_	0	0	0	1300	0
g_K,1.6_	0	0	0	800	0
g_Ca_	1	1	1	1	1
g_K,Ca_	0.1	0.1	0.1	0.1	0.1
g_L_	0.1	0.1	0.1	0.1	0.1

Values are based on Fohlmeister et al.^38^ with minor modifications. Values are given in mS/cm^2^.

### Quantification and statistical analysis

Recorded data were loaded and analyzed in MATLAB (Mathworks). Spike timing was detected as the depolarization (positive) peak at least crossing −25 mV and having a minimum depolarization rate of 25 mV/ms as spikes at small depolarization rates were shown not to be propagated along the axon of RGCs.^15^ Firing rate was computed by pooling responses from multiple trials (≥3) and subsequent convolution with a 50-ms sliding window.

Statistical analyses were performed in MATLAB (Mathworks) or Python. We used a two-sample *t* test or Wilcoxon rank-sum test for comparison of two groups depending on the distribution of the data. For comparison of multiple groups One-way ANOVA used Tukey’s honestly significant difference (HSD) post hoc test. Significance levels were set as follows: ∗*p* < 0.05, ∗∗*p* < 0.01, and ∗∗∗*p* < 0.001. Numerical values are presented as mean ± one standard error or deviation.

Principal component analysis (PCA) and k-means clustering were performed in MATLAB (Mathworks). PCA was performed on 19 parameters extracted from electrophysiological recordings: capacitance (pF), input resistance (MOhm), peak firing rate (Hz), maximum sustained firing rate (Hz), maximum rebound firing rate (Hz), maximum rebound firing rate timing (ms), break amplitude (pA), break voltage (mV), sag (mV), duration of rebound firing (ms), current threshold (pA), voltage threshold (mV), spike amplitude (mV), spike width (ms), maximum rate of hyperpolarization (mV/ms), maximum rate of depolarization (mV/ms), duration AIS to soma (ms), maximum rate of depolarization AIS (mV/ms) and onset rapidness (ms^−1^). Similarity between experimenter-based cell type classification and clustering based on intrinsic electrical properties was determined by the Adjusted Rand Index with a value of 1 indicating a perfect match.^46^
