# Supplementary material for: Intrinsic electrical diversity of PV+ retinal ganglion cells
Source: iScience. 2026 Aug 13;29(8):116842. doi: 10.1016/j.isci.2026.116842 (PMC13495337; doi:10.1016/j.isci.2026.116842)
Supplement: Supplementary file 1 [file mmc1.pdf]

iScience, Volume *29*

Supplemental information

Intrinsic electrical diversity of PV$^+$ retinal ganglion cells

Viktória Király, Günther Zeck, and Paul Werginz

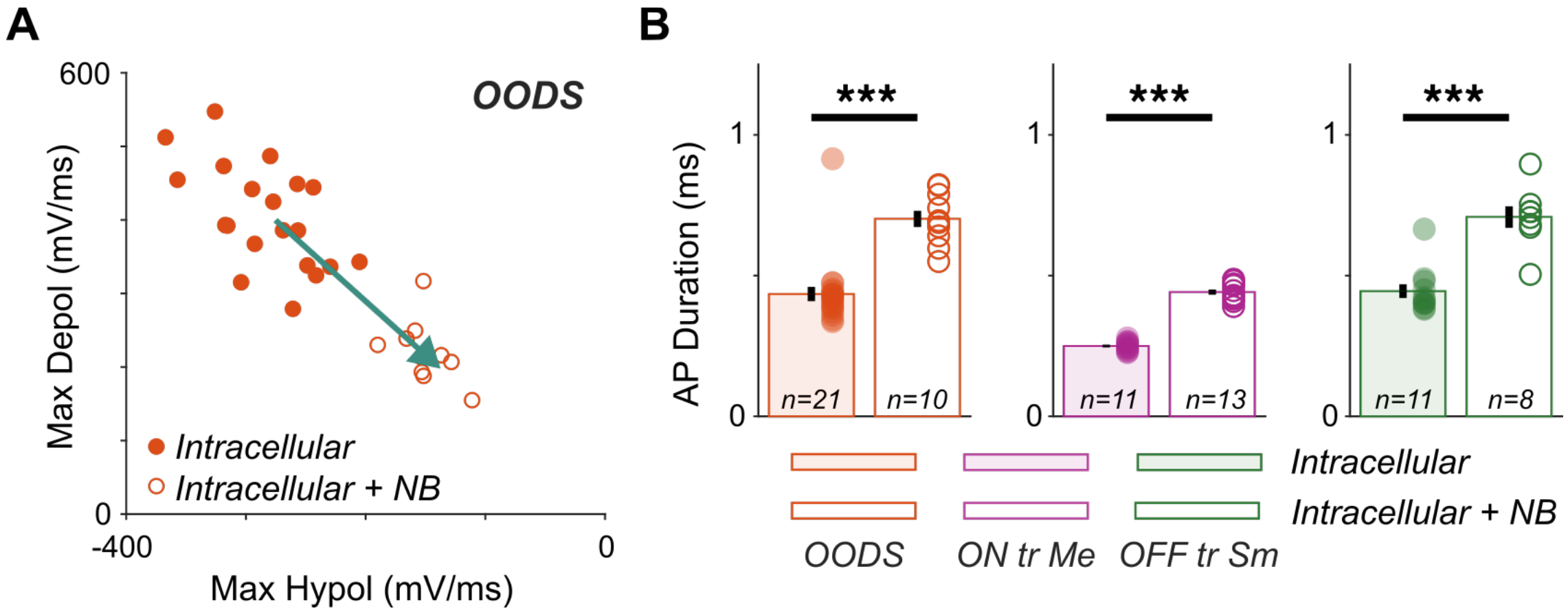

Figure S1. Effects of intracellular Neurobiotin filling on action potential dynamics in PV^{+} RGCs

(A) Comparison of maximum depolarization rate versus maximum repolarization rate in RGCs filled with standard intracellular solution, either with or without Neurobiotin. Each circle indicates a recording from single OODS RGC, the green arrow indicates a shift in maximum rate of de- and hyperpolarization when Neurobiotin was present in the intracellular solution.

(B) Bar plots comparing action potential duration for OODS (left, Wilcoxon rank sum test), ON tr Sm (middle, t-test), and OFF tr Sm (right, Wilcoxon rank sum test) RGCs under the two conditions (with and without Neurobiotin). Data are represented as mean +/- SEM, *** $p<0.001$.
